# Integrated region-specific modeling of H5 avian influenza in Asia using ENSO-based forecasts

**DOI:** 10.1016/j.onehlt.2026.101322

**Published:** 2026-01-07

**Authors:** Yinghui Su, Ruoxuan Wu, Pengfei Liu, Zhichao Li, Juan Pu, Lu Wang

**Affiliations:** a State Key Laboratory of Veterinary Public Health and Safety, College of Veterinary Medicine, China Agricultural University, Beijing 100193, China; b Key Laboratory for Resource Use and Environmental Remediation, Institute of Geographic Sciences and Natural Resources Research, Chinese Academy of Sciences, Beijing 100101, China

**Keywords:** El Niño-southern oscillation, Multivariate ENSO index, H5 highly pathogenic avian influenza, Generalized additive models, Climate-informed early warning, One Health surveillance

## Abstract

Highly pathogenic avian influenza (HPAI), particularly of the H5 subtype, remains a persistent threat to poultry, wildlife, and public health across Asia. This study quantifies the influence of the El Niño–Southern Oscillation (ENSO), using the Multivariate ENSO Index (MEI) as the primary predictor, on the climate-driven dynamics of H5 HPAI through region- and host-stratified generalized additive models (GAMs). Seven region–host strata across Asia were modeled separately, revealing pronounced heterogeneity in event frequency. A clear negative correlation with MEI was identified in domestic poultry across East and South Asia, where higher MEI values, corresponding to El Niño conditions, were linked to reduced event frequencies. In contrast, wild bird populations in East and South Asia displayed irregular, multimodal response patterns to MEI, suggesting phase-specific sensitivities to climate variability. A recurrent neural network (RNN) was further employed to forecast MEI trends, which were then incorporated into the GAMs to predict event dynamics. The forecasts highlighted continued epidemic pressure in East Asia's wild birds, in contrast to stable or declining trends elsewhere. Given the zoonotic potential of H5 viruses, these climate-informed risk forecasts could help inform timely interventions to prevent animal-to-human transmission and support integrated One Health preparedness frameworks. This integrative statistical–deep learning framework offers valuable support for short-term early warning and regionally targeted prevention strategies for H5 HPAI preparedness across Asia.

## Highlights

• Region- and host-stratified GAMs revealed heterogeneous effects of ENSO on H5 HPAI event frequency across Asia.
• Higher MEI values (El Niño conditions) were negatively correlated with poultry event counts in East and South Asia.
• RNN-based MEI forecasts were integrated into the GAM framework for forward-looking riskprediction.
• Forecasts indicated continued epidemic pressure in East Asia's wild birds, contrasting with stable or declining trends in other regions.
• The integrative framework supports One Health early warning systems and targeted cross-sector prevention strategies.

## 1 Introduction

Avian influenza is a contagious disease of birds caused by influenza A viruses belonging to the *Orthomyxoviridae* family. The world is currently facing an unprecedented crisis of highly pathogenic avian influenza (HPAI), with profound ecological and economic consequences. Since 2005, HPAI has resulted in the death or culling of over 633 million poultry worldwide, with an unprecedented peak of 146 million cases reported in 2022 alone [1]. It is important to note that from 2019 to 2022, HPAI outbreaks were not limited to poultry but also significantly affected wild bird populations, with widespread outbreaks reported across multiple continents. Despite temporal and regional variation, HPAI continues to circulate among both domestic poultry and wild birds, highlighting the enduring and dynamic nature of this global threat.

Of particular concern is HPAI caused by viruses of the H5 subtype, such as H5N1 and H5N8, which pose severe threats to avian populations and, more critically, to public health due to their zoonotic potential [2]. This concern has intensified with recent findings that emerging H5N1 strains, specifically those of the currently predominant clade 2.3.4.4b, can evade immune responses induced by prior infections or vaccinations, thereby increasing the risk of avian-to-human spillover [3]. The ongoing threat is further highlighted by a recent World Organization for Animal Health (WOAH) risk assessment, which identified high risks of wildlife spillover events in Asia and pointed to critical gaps in surveillance, communication, and resource allocation across multiple countries. This underscores the urgent need for enhanced cross-sectoral coordination to improve early detection of zoonotic threats [4].

From 2019 to 2022, HPAI H5 viruses spread widely among avian populations in Europe, Africa, and Asia, leading to a dramatic increase in global avian cases—from 0.343 million to 25.19 million [5]. In particular, East, South, and Southeast Asia have experienced extensive outbreaks in recent years. Japan recorded its worst epidemic during 2022–2023, with 17.7 million poultry culled, while countries such as China and South Korea have reported sustained H5 virus circulation in poultry populations (Business [2,[5], [6], [7]]). Between 2003 and 2024, the World Health Organization confirmed 889 human H5N1 infections and 463 fatalities globally—all reported in Asia and Africa—underscoring the significant public health risks in these regions [8].

The epidemiology of H5 avian influenza is highly complex, exhibiting substantial spatial and temporal variability. Growing evidence suggests that climatic factors—particularly those associated with global climate change—play a pivotal role in shaping outbreak dynamics and influencing host migration patterns [[9], [10], [11]]. Among these, the El Niño–Southern Oscillation (ENSO) is a quasi-periodic climate phenomenon driven by coupled ocean–atmosphere interactions in the equatorial Pacific and widely recognized as a key ecological driver due to its global reach. ENSO alternates between two phases: El Niño, marked by sea surface warming in the central and eastern Pacific, and La Niña, by cooling in the same regions. These shifts can disrupt atmospheric circulation and alter local environmental conditions such as temperature, humidity, and precipitation [12].

ENSO-driven climate anomalies may substantially influence the occurrence and seasonal dynamics of avian influenza by altering environmental conditions that affect viral persistence and host behavior. In China, multiple H5 subtypes (e.g., H5N2, H5N8, H5N6, H5N5) co-circulate and their seasonal activities appears to align with climatic variability [13]. ENSO is widely regarded as a key proxy for large-scale climate variability in infectious disease research [14]. While links between ENSO and influenza in humans have been explored [15,16], studies specifically examining its influence on H5 avian influenza remain scarce.

Although previous studies have highlighted the role of local climatic factors, such as temperature, humidity, and precipitation, in shaping H5 influenza activity [[22], [23], [24], [25]], systematic analyses incorporating large-scale climate drivers such as ENSO remain absent in the context of avian influenza. While several studies have examined ENSO-related effects on human influenza dynamics, key modeling components including variable selection, parameter tuning, and ecological stratification are rarely implemented, limiting the capacity to capture complex and nonlinear interactions between climate anomalies and disease risk [24,26].

To address these limitations, this study aims to quantitatively model the relationship between ENSO and H5 HPAI event occurrences across Asia using generalized additive models (GAMs). Given the ecological and epidemiological heterogeneity of the region, region-specific models were developed to accommodate spatial variability in epidemic dynamics. The Multivariate ENSO Index (MEI) was selected as the primary explanatory variable due to its integrated representation of multiple atmospheric and oceanic conditions, including sea surface temperature, sea-level pressure, wind patterns, and humidity, thereby offering a comprehensive and temporally continuous indicator of ENSO events. This modeling framework enables the quantification of ENSO's influence on epidemic risk in a spatially and temporally explicit manner. To enhance model robustness and predictive validity, a systematic workflow was adopted, including model selection, smoothing parameter optimization, and performance evaluation through appropriate metrics and validation techniques. These steps were essential to identify the optimal model for each subregion and to ensure stability and generalizability across diverse ecological contexts.

By incorporating an integrative climate index into region-specific models, this study provides new insights into how large-scale climate variability may shape the temporal dynamics of H5 HPAI occurrence across Asia. Built upon a systematically optimized and rigorously validated modeling framework, the findings offer a quantitative basis for understanding and anticipating epidemic trends, supporting the development of climate-informed early warning systems and risk mitigation strategies tailored to different subregions. Ultimately, this work contributes to enhancing predictive capacity in settings where persistent epidemics pose ongoing threats to both animal health and public safety. Furthermore, by identifying periods of elevated outbreak risk among poultry and wild birds, this framework can support proactive interventions to prevent zoonotic spillover, thereby strengthening One Health strategies that protect both animal and human populations.

## 2 Materials and methods

### 2.1 Epidemiological and climate data

#### 2.1.1 H5 highly pathogenic avian influenza (HPAI) event data

H5 HPAI data were obtained from the Global Animal Disease Information System (EMPRES-i, https://empres-i.apps.fao.org), maintained by the Food and Agriculture Organization (FAO) of the United Nations. All laboratory-confirmed H5 HPAI event records reported in Asia from January 2002 to February 2025 were extracted, based on data retrieved in mid-March 2025. To minimize potential duplication arising from multi-level or cross-border reporting, the EMPRES-i records were further harmonized using country, observation date, geographic coordinates, and host type to identify and remove any apparent duplicate events. Each record contains the affected host species, observation date, and geographic attributes such as region and country.

#### 2.1.2 Multivariate ENSO index (MEI) data

Climate data were obtained from the National Oceanic and Atmospheric Administration (NOAA, https://origin.cpc.ncep.noaa.gov), specifically the Multivariate ENSO Index version 2 (MEI.v2), hereafter referred to as MEI. This index integrates five key atmospheric and oceanic variables over the tropical Pacific: sea-level pressure (SLP), sea surface temperature (SST), surface zonal and meridional winds, and outgoing longwave radiation (OLR).

MEI records have been released monthly since January 1979, with each value representing a bimonthly average. For ENSO trend visualization and forecasting, the full historical MEI series was used. For modeling the relationship between MEI and H5 HPAI event occurrences, we extracted data from January 2002 to February 2025 to align with the temporal span of the avian influenza dataset. To facilitate monthly alignment in the models, each MEI value was assigned to the second month of its corresponding bimonthly period (e.g., the MEI for January–February was treated as the value for February). This adjustment ensured temporal synchronization between the climatic predictor and monthly event counts. MEI values were standardized and ranged approximately from −3 to +3, with positive and negative values corresponding to El Niño and La Niña conditions, respectively. In this study, positive and negative MEI values were used for descriptive purposes only in an exploratory phase-based comparison, whereas all main analyses treated MEI as a continuous predictor.

### 2.2 Analytical framework and modeling strategy

This study employed a four-step analytical framework integrating hypothesis testing, statistical modeling, and forecasting to investigate the influence of climate variability on H5 HPAI epidemics across Asia. Except for MEI forecasting, all analyses were conducted separately for each region–host stratum, defined by the cross-classification of five Asian subregions (East, Southeast, South, West, and Central) and two host types (domestic poultry and wild birds). Owing to insufficient sample sizes, three strata—Southeast Asia–Wild, Central Asia–Wild, and Central Asia–Domestic—were excluded from the analysis. MEI forecasting was performed in Python (v 3.10.14) using the TensorFlow framework, while all other procedures, including the Generalized Additive Models (GAMs) fitted using the mgcv package, were implemented in R (v 4.3.3).

#### 2.2.1 Phase-based event comparison

Permutation tests were applied to evaluate whether the number of H5 HPAI event occurrences differed significantly between El Niño and La Niña phases. Traditional parametric and rank-based tests (e.g., *t*-test and Wilcoxon rank-sum test) were not used, as their underlying assumptions—normality and distributional symmetry—were violated. For each region–host stratum, 10,000 Monte Carlo permutations were conducted to assess differences in both the mean and median event counts between the two ENSO phases. Statistical significance was determined based on two-sided *p*-values derived from the empirical null distributions. All permutation-test p-values were computed using two-sided alternatives.

#### 2.2.2 Modeling MEI effects on event counts with generalized additive models (GAM)

##### 2.2.2.1 Model specification and selection

To identify the optimal specification of ENSO effects and long-term trends on avian influenza event counts, we formulated six candidate generalized additive models (GAMs), each differing in the functional forms applied to MEI (linear, quadratic, or smooth) and time (linear or smooth) variables.

Model 1: $\mathrm{logEY}=\beta_{0}+\beta_{1}\mathrm{MEI}+\sum_{m=2}^{12}\beta_{m}I_{\text{Month}=m}+\beta_{13}\mathrm{time}+\varepsilon .$

Model 2: $\mathrm{logEY}=\beta_{0}+\beta_{1}\mathrm{MEI}+\beta_{2}{\mathrm{MEI}}^{2}+\sum_{m=2}^{12}\beta_{m+1}I_{\text{Month}=m}+\beta_{14}\mathrm{time}+\varepsilon$

Model 3: $\mathrm{logEY}=\beta_{0}+f\left(\mathrm{MEI}\right)+\sum_{m=2}^{12}\beta_{m-1}I_{\text{Month}=m}+\beta_{12}\mathrm{time}+\varepsilon$

Model 4: $\mathrm{logEY}=\beta_{0}+\beta_{1}\mathrm{MEI}+\sum_{m=2}^{12}\beta_{m}I_{\text{Month}=m}+f\left(\mathrm{time}\right)+\varepsilon$

Model 5: $\mathrm{logEY}=\beta_{0}+\beta_{1}\mathrm{MEI}+\beta_{2}{\mathrm{MEI}}^{2}+\sum_{m=2}^{12}\beta_{m+1}I_{\text{Month}=m}+f\left(\mathrm{time}\right)+\varepsilon$

Model 6: $\mathrm{logEY}=\beta_{0}+f_{1}\left(\mathrm{MEI}\right)+\sum_{m=2}^{12}\beta_{m-1}I_{\text{Month}=m}+f_{2}\left(\mathrm{time}\right)+\varepsilon$

Here, Y denotes the monthly event count; $I_{\text{Month}=m}$ is a dummy variable for months (January as the reference); time is defined as the number of months since January 2002 (e.g., January 2002 = 1, February 2002 = 2, etc.); $f\left(∙\right),f_{1}\left(∙\right),\text{and}\;f_{2}\left(∙\right)\;$denote spline-based smooth functions; and ε is the error term following either a Poisson or negative binomial distribution, depending on the degree of overdispersion. The month and time variables account for seasonal and long-term trends, respectively. Monthly dummy variables were employed to capture potential discontinuities or abrupt changes in seasonal effects that splines might otherwise smooth out, and to avoid imposing artificial periodicity in strata where seasonal information is sparse.

For the two strata with limited sample sizes (South Asia–Wild, *n* = 40; West Asia–Wild, *n* = 33), monthly dummy variables were replaced with a seasonal factor—Spring (January–March), Summer (April–June), Fall (July–September), and Winter (October–December)—to reduce model complexity.

Overdispersion was assessed separately within each region–host stratum using Pearson residuals from an initial Poisson GAM. Specifically, the dispersion parameter was estimated as the ratio of the sum of squared Pearson residuals to the residual degrees of freedom. Poisson models were retained when the dispersion parameter was ≤ 1; otherwise, negative binomial models were applied to accommodate extra-Poisson variation.

Each candidate model was evaluated using 1000 bootstrap resamples, and the model with the lowest Akaike Information Criterion (AIC) was selected for subsequent spline basis specification and parameter tuning.

##### 2.2.2.2 Spline basis selection and smoothing parameter tuning

To flexibly capture nonlinear effects and optimize model smoothness, two types of spline basis functions were compared: cubic regression splines (CRS) and thin plate regression splines (TPRS). CRS ensures continuity of the fitted function and its first and second derivatives, while TPRS minimizes the overall curvature of the smooth term and allows adaptive knot placement, making it particularly suitable for multidimensional smoothing.

For most strata, the number of knots for the *time* smooth term was varied from 5 to 40 in increments of 5 (i.e., k = 5, 10, …, 40). For strata with small sample sizes (South Asia–Wild, *n* = 40; West Asia–Wild, *n* = 33), a narrower grid from 5 to 10 in steps of 1 was used to reduce the risk of overfitting and enhance model stability.

Model performance was evaluated via Monte Carlo cross-validation with 1000 replications. In each replicate, the dataset was randomly split into 80 % training and 20 % test sets. GAMs were fitted to the training data, and predictive accuracy was assessed on the test set using root mean squared error (RMSE):

$$\mathrm{RMSE}=\sqrt{\frac{1}{n}\sum_{i=1}^{n}{\left(y_{i}-{\hat{y}}_{i}\right)}^{2}}$$

where $y_{i}$ and ${\hat{y}}_{i}$ denote the observed and predicted event counts, respectively. The average RMSE across the 1000 replications was computed for each combination of spline basis and knot number, and the configuration with the lowest mean RMSE was selected as the final model.

##### 2.2.2.3 Model diagnostics

Model diagnostics were performed to assess the adequacy and robustness of the final model. Residuals analyses were conducted to evaluate distributional assumptions, to identify potential outliers or patterns of heteroscedasticity, and to examine temporal dependence using the ACF and PACF of the GAM residuals. Fitted values were compared against observed counts to assess overall model performance. The complexity of smooth terms was reviewed based on estimated effective degrees of freedom, and the plausibility of estimated nonlinear relationships was assessed through visual inspection of the smooth functions. These diagnostic procedures were used to verify assumption validity, evaluate model flexibility, and minimize the risk of overfitting.

#### 2.2.3 Forecasting MEI using deep learning

To forecast future MEI values, five deep learning models were evaluated: multilayer perceptron (MLP), one-dimensional convolutional neural network (CNN), recurrent neural network (RNN), long short-term memory network (LSTM), and hybrid CNN–LSTM model. The complete MEI time series was reformatted into a supervised learning structure using a sliding window approach. A sliding-window design was used, in which each training sample consisted of the preceding 12 months of MEI values to forecast the subsequent 6 months. This window length provided sufficient short-term temporal information while avoiding over-parameterization given the limited historical record.

The dataset was split into training and validation sets in an 80:20 ratio and normalized prior to training. Each model was trained for 200 epochs. Key hyperparameters (e.g., neuron counts, kernel sizes, pooling strategies) were standardized across models to ensure comparability. To preserve the temporal structure, data shuffling was avoided.

Model performance was evaluated on the validation set, with root mean squared error (RMSE) serving as the primary selection metric. In cases where models exhibited nearly identical RMSE values, the coefficient of determination ($R^{2}$) was used as a secondary criterion to assess explanatory power. The final model was selected based on validation performance and applied to obtain MEI estimates for the subsequent six months.

#### 2.2.4 Prediction of H5 HPAI event counts in Asia

The MEI forecasts generated by the optimal deep learning model were used as an explanatory variable in the best-fitting GAMs described in Section 2.2.2. For consistency with operational short-range forecasting, the MEI predictions were incorporated into the GAM as deterministic point estimates. For each region–host stratum, the corresponding GAM was applied to estimate the expected number of H5 HPAI events over the next six months. This integrative modeling framework enabled forward-looking estimation of avian influenza risk under projected climate conditions.

## 3 Results

### 3.1 Descriptive overview of MEI and H5 HPAI outbreaks

#### 3.1.1 MEI time series and ENSO phase distribution

**Fig. 1:**
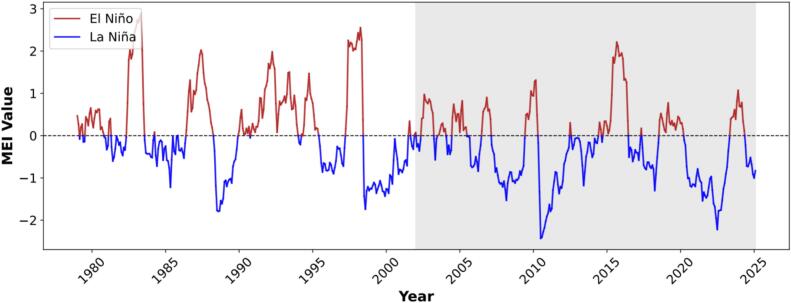
Temporal variation in the Multivariate ENSO Index (MEI), January 1979–February 2025. Positive and negative values indicate El Niño (red) and La Niña (blue) conditions, respectively. The shaded period (January 2002–February 2025) corresponds to the analysis window used for modeling H5 HPAI outbreaks. (For interpretation of the references to colour in this figure legend, the reader is referred to the web version of this article.)

Fig. 1 illustrates the temporal dynamics of the Multivariate ENSO Index (MEI) from January 1979 to February 2025, with positive (red) and negative values (blue) representing El Niño and La Niña phases, respectively. The time series highlights the cyclical nature of ENSO, with several prominent El Niño episodes (e.g., 1982–1983, 1997–1998) and La Niña episodes (e.g., 2010–2011, 2020–2023).

The analysis period relevant to this study spans from January 2002 to February 2025, during which MEI exhibited multiple ENSO transitions. Within this period, 235 months were classified as El Niño, 301 as La Niña, and 4 as neutral. Neutral months (MEI = 0) accounted for only ∼0.6 % of all months and were therefore considered negligible for phase-based comparisons. These interannual climate fluctuations provide the temporal foundation for evaluating the potential influence of ENSO on H5 HPAI outbreaks across Asia.

#### 3.1.2 Spatiotemporal distribution of H5 HPAI events

Fig. S1 illustrates the distribution of reported H5 HPAI events across five Asian subregions, stratified by host type (domestic poultry and wild birds). Southeast Asia exhibited the highest overall number of events, primarily driven by cases in domestic poultry, whereas Central Asia had the lowest reporting frequency.

Wild bird-associated events were predominantly reported in East Asia (1322 events, 85.9 %), followed by West Asia (108 events, 7.0 %) and South Asia (90 events, 5.9 %). In contrast, Southeast Asia (12 events, 0.8 %) and Central Asia (6 events, 0.4 %) reported only a small number of events. Domestic poultry-associated events were most frequently reported in Southeast Asia (13,407 events, 70.5 %), with fewer occurrences in East Asia (3704 events, 19.5 %), South Asia (1364 events, 7.2 %), West Asia (537 events, 2.8 %), and Central Asia (14 events, 0.1 %).

These patterns underscore pronounced geographic and host-type heterogeneity in H5 HPAI event reporting, providing a critical basis for the region- and host-specific modeling strategies adopted in subsequent analyses.

### 3.2 Phase-based comparison of H5 HPAI event counts

**Fig. 2:**
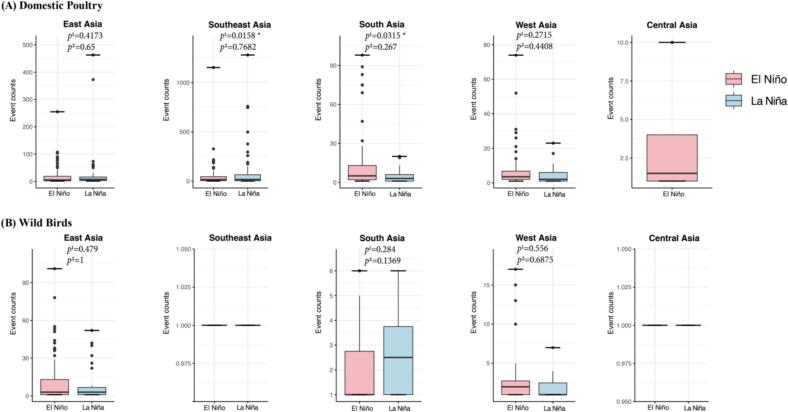
Comparison of monthly H5 HPAI event counts between El Niño and La Niña phases across five Asian subregions. Panel A: domestic poultry; Panel B: wild birds. Asterisks (*) indicate statistically significant differences (*p* value <0.05) based on permutation tests. Two tests were conducted per stratum: $p^{1}$ and $p^{2}$ for mean and median differences, respectively.

To preliminarily evaluate the influence of ENSO phases on avian influenza dynamics, permutation tests were conducted to compare monthly H5 HPAI event counts between El Niño and La Niña phases across different region–host strata. As shown in Fig. 2, results are presented separately for domestic poultry (Panel A) and wild birds (Panel B). Permutation tests were not conducted for three strata—Southeast Asia–Wild (*n* = 12), Central Asia–Domestic (*n* = 14), and Central Asia–Wild (*n* = 6), because their extremely limited sample sizes did not permit statistically meaningful comparisons or valid statistical inference

For strata with sufficient observations, two permutation tests were performed: one comparing mean event counts ($p^{1}$) and the other comparing medians ($p^{2}$). The statistical significance level was set at 0.05. Significant differences in mean counts were observed in domestic poultry populations in Southeast Asia ($p^{1}$ = 0.0158) and South Asia ($p^{1}$ = 0.0315), suggesting that El Niño and La Niña phases may influence outbreak intensity in these areas. However, corresponding median-based tests were not significant ($p^{2}$ = 0.7682 and 0.2670, respectively), indicating that the significant mean differences were likely driven by sporadic outbreak surges in a few high-count months rather than consistent phase-wide shifts. In all other region–host combinations, including domestic poultry in East and West Asia and wild birds in East, South, and West Asia, no significant differences were detected under either test.

These findings provide preliminary evidence of potential ENSO-related variations in H5 HPAI occurrence, particularly in domestic poultry populations in Southeast and South Asia. To capture the full gradient of climatic variability, subsequent models incorporated MEI as a continuous variable rather than a binary phase indicator, allowing for more efficient use of climate information and more sensitive detection of its association with disease risk.

### 3.3 Estimation of MEI effects on event counts based on GAMs

Generalized additive models (GAMs) with a negative binomial distribution were fitted to all region–host strata, except for South Asia–Wild birds, where overdispersion was not detected and a Poisson family was therefore adopted. Model selection procedures identified Model 6 as the best-fitting configuration for East Asia–Domestic poultry, East Asia–Wild birds, South Asia–Domestic poultry, South Asia–Wild birds, West Asia–Domestic poultry, and West Asia–Wild birds. In contrast, Model 4 provided the best fit for Southeast Asia–Domestic poultry. Details regarding the selected spline basis functions and the number of knots used in each model are provided in Supplementary Table S1.

**Fig. 3:**
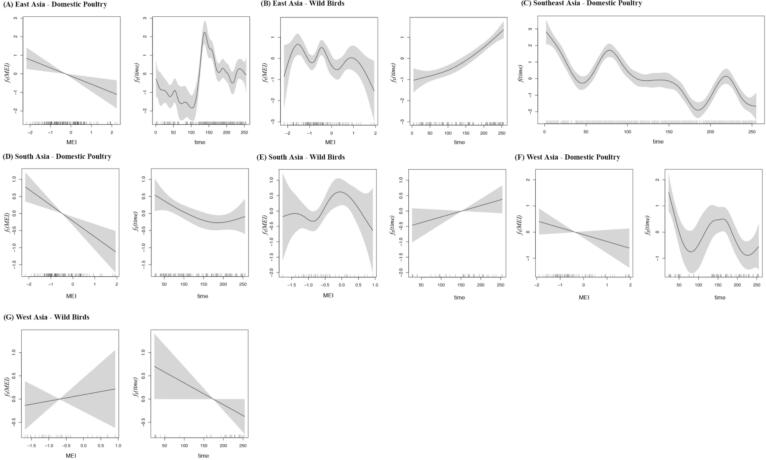
Estimated effects of MEI and time derived from the optimal GAMs for seven Asian region–host strata, with 95 % Bayesian credible intervals. Each panel corresponds to a specific region–host combination: (A) East Asia–Domestic Poultry, (B) East Asia–Wild Birds, (C) Southeast Asia–Domestic Poultry (Model 4: MEI modeled as a linear term; both the linear MEI effect and the smooth time effect are shown), (D) South Asia–Domestic Poultry, (E) South Asia–Wild Birds, (F) West Asia–Domestic Poultry, (G) West Asia–Wild Birds.

Fig. 3 presents the estimated effects of MEI and time across the seven region–host strata. In most cases, both variables were modeled using smooth terms; however, in the case of Southeast Asia–Domestic poultry, MEI was modeled as a linear term instead. The resulting curves reveal substantial variation in both magnitude and functional form, reflecting the climatic and temporal heterogeneity across regions and host types.

Across the seven region–host strata, MEI was associated with different patterns in H5 HPAI event counts. In domestic poultry populations from East, South and West Asia, MEI exhibited a broadly decreasing trend (Fig. 3A, D, F), suggesting that higher MEI values—corresponding to El Niño conditions—may be associated with lower event frequencies. In contrast, wild bird populations in East and South Asia displayed irregular, non-monotonic response curves with alternating peaks and troughs (Fig. 3B, E), implying phase-specific sensitivities to both El Niño and La Niña conditions. Among wild birds in West Asia, the MEI effect remained close to zero but showed a slight upward drift across the index range (Fig. 3G). As MEI was not retained as a smooth term in the model for domestic poultry in Southeast Asia, a supplementary linear-term analysis indicated no substantial association between MEI and outbreak frequency (Fig. 3C).

The estimated time effects also revealed diverse temporal dynamics. The domestic poultry in East Asia exhibited a pronounced mid-period peak, indicative of a unimodal temporal pattern (Fig. 3A). Wild birds in East and South Asia showed sustained upward trends, pointing to increasing event counts in recent years (Fig. 3B, E). In contrast, domestic poultry in South Asia and wild birds in West Asia followed gradual downward trajectories, with the former showing a modest recovery near the end of the period (Fig. 3D, G). Domestic poultry in both Southeast and West Asia displayed downward trajectories superimposed on broader temporal fluctuations, suggesting declining trends embedded within irregular seasonal or interannual variability (Fig. 3C, F).

In addition to the smooth effect plots, model fitting performance was evaluated by comparing the GAM-estimated event counts to observed data. These results are presented in Fig. 5A–5G. In each panel, the orange line represents the fitted event counts generated by the optimal GAMs over the historical observation period, while the black line denotes the actual reported H5 HPAI events. The close alignment between the fitted and observed trajectories across most strata supports the validity of the models in capturing temporal variation. In general, the fitted values closely tracked the seasonal and interannual patterns of observed events, although slight underestimations were noted during short-lived outbreak peaks. Such deviations are common in smooth-based models, which prioritize capturing overall structural trends over highly localized spikes.

Overall, the GAM-based analyses reveal heterogeneous and region-specific effects of MEI and time on H5 HPAI event dynamics, shaped by differences in host type and geographic context. These results provide a robust statistical foundation for understanding climate-sensitive patterns in the frequency of H5 HPAI occurrences across Asia, and offer a basis for short-term projections of disease activity in response to climate variability.

### 3.4 Forecasting MEI with deep learning

Five deep learning models, including MLP, CNN, RNN, LSTM, and CNN–LSTM, were evaluated for their ability to forecast the Multivariate ENSO Index (MEI). Based on multiple evaluation metrics, including RMSE and R^2^ (Fig. S2), the RNN model demonstrated the best overall performance and was selected as the optimal model for future MEI prediction. The training and validation loss curves (Fig. S3) further confirmed the stability and convergence of the RNN model over 200 epochs, supporting its reliability in time series forecasting.

**Fig. 4:**
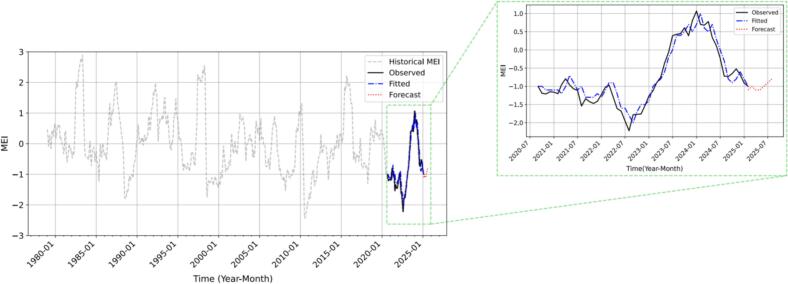
Forecasted MEI values from March to August 2025 using the RNN model. The gray dashed line represents the complete historical MEI data, the black line denotes observed values from September 2020 to February 2025, the blue line indicates predictions over the same period, and the red line depicts the six-month forecast from March to August 2025. The inset provides an enlarged view of the September 2020–February 2025 interval to highlight prediction accuracy and temporal alignment. (For interpretation of the references to colour in this figure legend, the reader is referred to the web version of this article.)

Fig. 4 displays the forecasting results, including the six-month projection of MEI values from March to August 2025 (Table S2). The predicted values show a gradual upward trend, which is consistent with the quasi-periodic nature of ENSO fluctuations. This pattern aligns with the results of the seasonal decomposition of the historical MEI series (Fig. S4), where recurring short-term cycles of 3–5 months (approximately 90, 122, and 153 days) were observed. The predicted MEI values range from −1.1 to −0.8 across the forecast window, indicating a gradual weakening of La Niña–like conditions.

### 3.5 Projection of future H5 HPAI event counts

To assess the impact of projected climate conditions on H5 HPAI occurrences, we applied the GAMs developed in Section 3.3 using the MEI values forecasted by the RNN model in Section 3.4. For each region–host stratum, the corresponding GAM was used to estimate monthly H5 HPAI event counts over a six-month forecast window from March to August 2025.

**Fig. 5:**
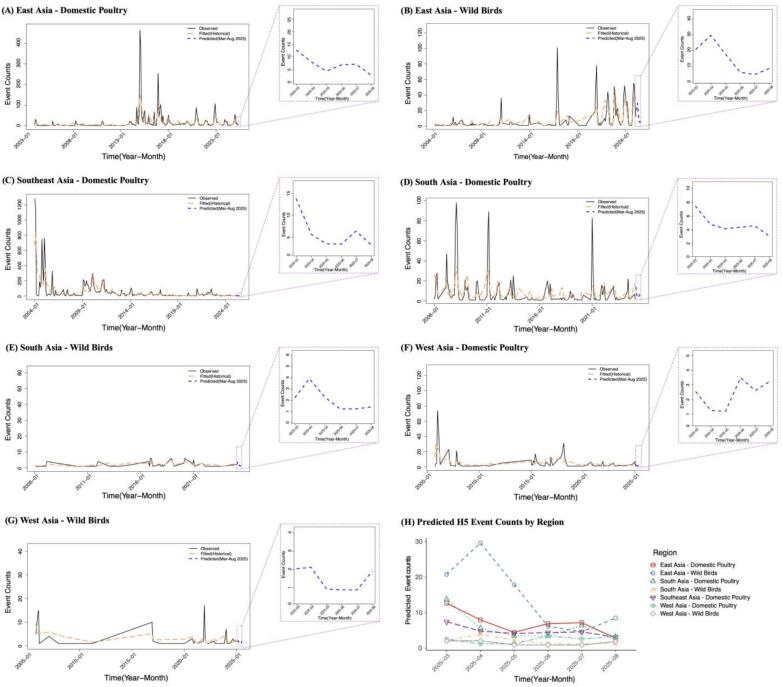
Fitting performance and six-month forecasts for seven region–host GAMs. Panels (A)–(G) show observed (black lines) and model-fitted (orange lines) H5 HPAI event counts for each region–host stratum. Insets within each panel display the six-month forecasts from March to August 2025 with 95 % confidence intervals. Specifically, the panels correspond to: (A) East Asia–Domestic Poultry, (B) East Asia–Wild Birds, (C) Southeast Asia–Domestic Poultry, (D) South Asia–Domestic Poultry, (E) South Asia–Wild Birds, (F) West Asia–Domestic Poultry, and (G) West Asia–Wild Birds. Panel (H) summarizes the projected outbreak trajectories across all strata over the forecast horizon.

Fig. 5 presents the six-month forecasts of H5 HPAI event counts across the seven region–host strata. Insets within Panels (A)–(G) show the predicted values and the corresponding 95 % confidence intervals. Panel (H) provides an aggregated summary of forecasted outbreak trajectories across all strata, highlighting variation in expected epidemic activity by host type and geographic region.

Wild birds and domestic poultry in East Asia, as well as domestic poultry in South Asia, exhibited short-term fluctuations superimposed on an overall declining trend. Notably, wild birds in East Asia showed the highest predicted event counts, with a sharp peak in April followed by a decline and a modest rebound in August. Domestic poultry in East Asia experienced a gradual decrease through June, accompanied by minor fluctuations thereafter. In South Asia, domestic poultry showed a brief decline in spring, followed by a moderate increase during early summer before stabilizing.

In contrast, the remaining four strata—domestic poultry in Southeast and West Asia, and wild birds in South and West Asia—maintained relatively low and stable levels throughout the forecast period. Among these, wild birds in West Asia consistently recorded the lowest predicted event counts.

Collectively, these forecasts reveal heterogeneous yet regionally consistent trends in H5 HPAI activity across Asia. Several region–host strata exhibited declining trajectories, aligning with seasonal expectations, while others maintained low but steady levels throughout the forecast period. By integrating predicted MEI values into region-specific GAMs, this modeling framework enabled forward-looking estimation of outbreak risk under projected climate conditions. These findings suggest that incorporating climate indices such as MEI into spatiotemporal prediction models enable more targeted and responsive disease risk assessment.

## 4 Discussion

### 4.1 Methodological scope and limitations

This study was designed to develop a statistical modeling framework to assess large-scale climate–disease associations rather than to infer biological or virological mechanisms. Specifically, we aimed to identify whether ENSO-related variability, represented by the Multivariate ENSO Index (MEI), provides statistically significant signals that could improve the predictive understanding of H5 HPAI occurrences across Asia. Accordingly, the analytical approach is statistical and predictive in nature, focusing on the detection of macro-level climatic associations rather than causal mechanisms of viral transmission.

To ensure statistical stability and comparability, wild bird outbreak records were aggregated into a single category. While species-level information was available for certain events, inconsistencies in documentation across time and reporting countries precluded reliable species-level stratification, which could otherwise introduce bias and uneven model representation. Likewise, although poultry vaccination programs are known to influence outbreak dynamics, systematic vaccination data were unavailable for the full study period, preventing their inclusion as covariates.

Similarly, virological factors—such as recent reductions in H5 HPAIV virulence in wild ducks—and ecological processes, including potential ENSO-driven changes in migratory flyways and population size at wintering sites, remain beyond the scope of this analysis due to the lack of regionally harmonized datasets. Nonetheless, by establishing a region- and host-specific statistical modeling framework, this study provides a foundation for integrating such ecological, virological, or management-related factors in future research.

### 4.2 Role of MEI and variability in climatic influence

This study revealed a consistent negative relationship between the Multivariate ENSO Index (MEI) and H5 HPAI event counts in domestic poultry populations from East and South Asia. Such a negative association aligns with previous research suggesting that El Niño conditions, typically reflected by elevated MEI values, may suppress avian influenza activity by altering temperature, humidity, and precipitation patterns [22,27]. Moreover, ENSO has been shown to exert profound influences on the climate of the Asia-Pacific region^**Error! Reference source not found.**^ [28], and has been correlated with shifts in bird migration timing and stopover sites in other studies [29,30]. Notably, warming-driven phenological changes in migratory birds have been reported across East, South, and Southeast Asia [31], suggesting that elevated MEI values may shorten the duration or frequency of contact between migratory wild birds and domestic poultry habitats, thereby reducing the risk of cross-species transmission.

In contrast, wild bird populations, particularly in East and South Asia, exhibited intermittent and non-monotonic associations with MEI. Multimodal response curves observed in these strata suggest that avian influenza activity may vary in association with specific ENSO phases or transitions. Such non-monotonic relationships may reflect the indirect pathways through which climate affects disease risk, particularly via migratory dynamics. These findings are supported by earlier studies reporting that ENSO events can influence migratory timing, stopover behavior, and resource availability, thereby introducing episodic transmission potential [32]. This pattern also highlights that under-reporting and spatial heterogeneity in wild bird surveillance can mask or distort climate–disease linkages.

We further note that regional studies in Europe have incorporated fine-scale climatic, land-use, and socio-economic data to predict poultry outbreaks, while a study in Bangladesh highlighted the central role of local market chains in driving H5N1 spread. By contrast, few studies to date have examined the role of global climate indices, such as ENSO, across multiple regions and host species. To bridge this knowledge gap, we reference regional investigations (e.g. [19]) and highlight that our study extends these findings by providing a global-scale, multi-region, and multi-host analysis of ENSO–avian influenza associations.

Overall, such variability highlights the limitations of relying solely on large-scale climate indices to forecast event patterns across diverse wild bird populations. This underscores the value of host- and region-specific modeling approaches, as implemented in our framework, which capture ecological and regional heterogeneity and provide more reliable predictive insights. Furthermore, the weak or negligible MEI effects observed in certain strata, such as wild birds in West Asia, suggest that climatic influence may be secondary in some settings, potentially reflecting the predominant role of anthropogenic or policy-driven factors rather than climatic variability alone.

### 4.3 Regional heterogeneity and predictive insights

The six-month predictions presented in Section 3.5 revealed marked heterogeneity in H5 HPAI event patterns across both host types and geographic regions. These differences carry important implications for risk surveillance and forecasting strategies. For instance, the elevated and fluctuating risk predicted for wild birds in East Asia underscores the need for intensified monitoring in migratory hotspots during spring and summer, when climate variability may heighten outbreak potential. Similar findings have been reported in regional studies, where climate variability was shown to alter the spatiotemporal distribution of avian influenza outbreaks. In contrast, the consistently low activity observed in wild birds from West Asia may reflect genuinely low incidence, underreporting, or ecological buffering, suggesting a different prioritization for surveillance efforts [17].

Transmission dynamics of avian influenza are influenced by multiple factors. In poultry populations, outbreaks are often driven by anthropogenic factors. Recent studies have shown that the spread of HPAI frequently follows poultry movement and vehicle-contact networks. For example, one network analysis estimated that production- and health-related vehicle movements accounted for nearly 30 % of H5N6 transmission between farms. Similarly, another study demonstrated that poultry outbreaks were correlated with human population density and trade-related drivers, whereas wild bird cases were more closely associated with natural environmental factors [18,19]. In our model, we incorporated smooth temporal trends and seasonal components to account for changes in control policies and surveillance practices over 2000–2025, an approach commonly applied in epidemiological modeling [20]. While this adjustment cannot fully remove all potential confounding effects, it serves to mitigate unmeasured variability.

In contrast, avian influenza transmission among wild birds exhibits distinct temporal and spatial patterns. Seasonal migrations across multiple flyways contribute to complex dynamics and justify the global analytical scope of our study. However, under-reporting remains a major challenge in wild bird surveillance, with considerable heterogeneity across both space and time. Such reporting biases can be misinterpreted as ecological or climatic risk factors. Global reviews indicate that wild bird surveillance efforts are patchy and non-standardized. For example, Machalaba et al. [17] reported that, although 119 countries conducted wild bird surveillance between 2008 and 2013, these efforts were sporadic, poorly coordinated, and often focused on specific subtypes. Some modeling studies have attempted to address this bias. For instance, Belkhiria et al. [21] used a pseudo-absence approach to adjust for under-reported wild bird influenza data, thereby improving risk estimation. Simple temporal and seasonal adjustments may not fully remove these biases, highlighting the need for cautious interpretation of wild bird results.

The region- and host-stratified modeling framework enabled the identification of strata requiring differentiated forecasting strategies. By capturing both climatic and ecological heterogeneity, this stratified approach facilitates more precise allocation of surveillance resources and refinement of early warning systems. Rather than applying uniform surveillance thresholds across heterogeneous regions, the findings support adaptive, context-sensitive strategies tailored to local epidemiological conditions and host ecology. Despite these remaining limitations in poultry (residual confounding) and wild bird data (under-reporting), the stratified modeling framework provides valuable insights into H5 HPAI risk patterns and can inform adaptive surveillance strategies.

### 4.4 Modeling contributions and future perspectives

Building on these findings, we developed a novel region-specific, host-stratified modeling framework that integrates neural network–based MEI forecasting with GAM-based event modeling, capturing climatic, spatial, and temporal complexity in the estimation of HPAI event risk. Unlike previous studies that focused on single-country or continental-scale patterns, our region-host models revealed diverse temporal trajectories and functional associations with MEI across Asia.

This framework is implemented through a systematic and reproducible pipeline, including stratified data filtering, exclusion of regions lacking sufficient data, candidate model selection, spline specification, residual diagnostics, and final predictive execution. This end-to-end process enhances model robustness and interpretability, enabling replication in other emerging or high-risk settings.

Building on this framework, further refinements could improve forecasting accuracy and lead time. Future extensions could incorporate distributed lag nonlinear models (DLNMs), which are well-suited to capturing delayed climate effects, particularly relevant for migratory species and seasonal epidemics. Additionally, integrating phylogeographic data could help trace the spatial spread of viral lineages across regions and host species, providing mechanistic insights into how climate-driven bird migration shapes transmission dynamics.

In parallel, integrating higher-resolution meteorological variables (e.g., temperature, precipitation, humidity) could complement MEI by capturing localized climate signals, especially in regions where large-scale indices may not fully reflect environmental variability. These enhancements would expand the utility of the modeling framework in supporting climate-informed early warning systems and targeted intervention strategies. This multi-scale modeling strategy integrating macro and local factors would provide a more comprehensive understanding of complex climate-ecology-disease transmission mechanisms. Similarly, vaccination data could be incorporated as covariates when systematically available across study regions.

Beyond methodological advancement, the broader public health relevance of this framework lies in its applicability to integrated surveillance. Furthermore, by integrating these predictive insights into One Health surveillance frameworks, public health authorities could better anticipate periods of elevated risk and proactively allocate resources to prevent cross-species transmission and protect vulnerable human populations. The continued circulation of H5 HPAI among domestic poultry and wild birds raises the risk of zoonotic spillover, particularly in settings with frequent animal–human contact, such as live bird markets, smallholder farms, and migratory bird stopover sites [33,34].

## 5 Conclusion

This study establishes a novel region- and host-stratified modeling framework that integrates deep learning–based ENSO forecasting with generalized additive models for predicting H5 HPAI risk across Asia. By capturing both climatic and ecological heterogeneity, the framework enables forward-looking assessments that are sensitive to regional and host-specific epidemic dynamics.

The results reveal a consistent negative relationship between MEI and event risk in domestic poultry from East and South Asia, while wild bird populations exhibit more complex, intermittent associations. These findings highlight the importance of stratified, data-driven modeling in disentangling climate–disease linkages and tailoring risk assessment.

Notably, the reproducible modeling pipeline developed in this study, including spanning data curation, model selection, and validation, provides a robust basis for adaptation in other settings. This flexible framework can be readily adapted to other climate-sensitive infectious diseases and geographic settings. Integrating ENSO forecasts into epidemiological models may provide valuable lead time for short-term risk warning systems, supporting timely surveillance and intervention planning under increasing climate variability. This integrative approach also supports a One Health perspective by recognizing the interconnected risks to animal and human health, emphasizing the need for coordinated cross-sector collaboration to strengthen zoonotic disease preparedness.

## CRediT authorship contribution statement

**Yinghui Su:** Writing – review & editing, Writing – original draft, Visualization, Validation, Methodology, Investigation, Formal analysis, Data curation, Conceptualization. **Ruoxuan Wu:** Data curation, Conceptualization. **Pengfei Liu:** Data curation, Conceptualization. **Zhichao Li:** Methodology, Conceptualization. **Juan Pu:** Project administration, Funding acquisition, Conceptualization. **Lu Wang:** Writing – review & editing, Validation, Supervision, Methodology, Funding acquisition, Formal analysis, Conceptualization.

## Declaration of competing interest

The authors declare that they have no known competing financial interests or personal relationships that could have appeared to influence the work reported in this paper.

## Data Availability

Data will be made available on request.

## Appendix A Supplementary data

Supplementary material Image 1
