# Supplementary material for: Integrated region-specific modeling of H5 avian influenza in Asia using ENSO-based forecasts
Source: One Health. 2026 Jan 7;22:101322. doi: 10.1016/j.onehlt.2026.101322 (PMC12818151; doi:10.1016/j.onehlt.2026.101322)
Supplement: Supplementary file 1 — Supplementary material [file mmc1.docx]

**Supplementary material**


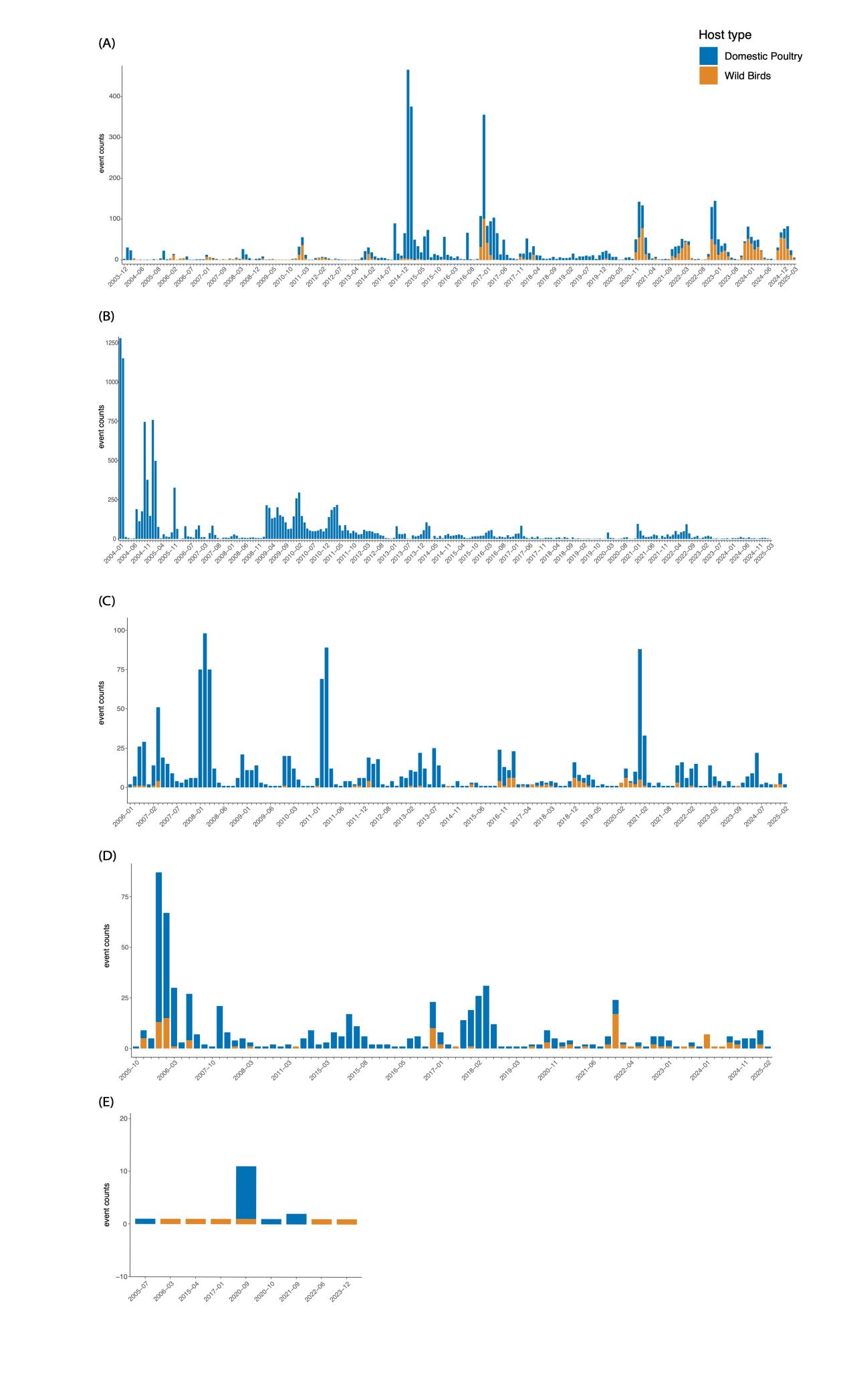


Figure S1 Temporal distribution of H5 HPAI event counts across five Asian subregions, stratified by host type. (A) East Asia; (B) Southeast Asia; (C) South Asia; (D) West Asia; (E) Central Asia. Bars represent monthly outbreak counts from January 2002 to February 2025, with blue and orange indicating domestic poultry and wild birds, respectively.


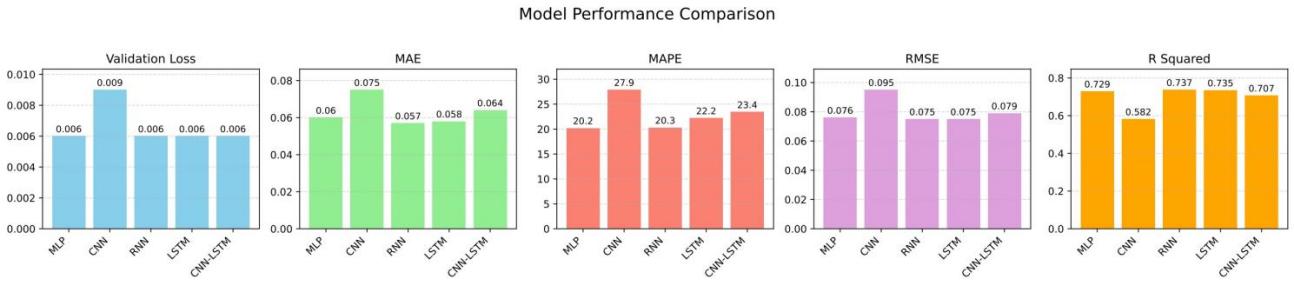


Figure S2 Comparison of model performance based on RMSE, MAE, MAPE, and R² on the validation set.


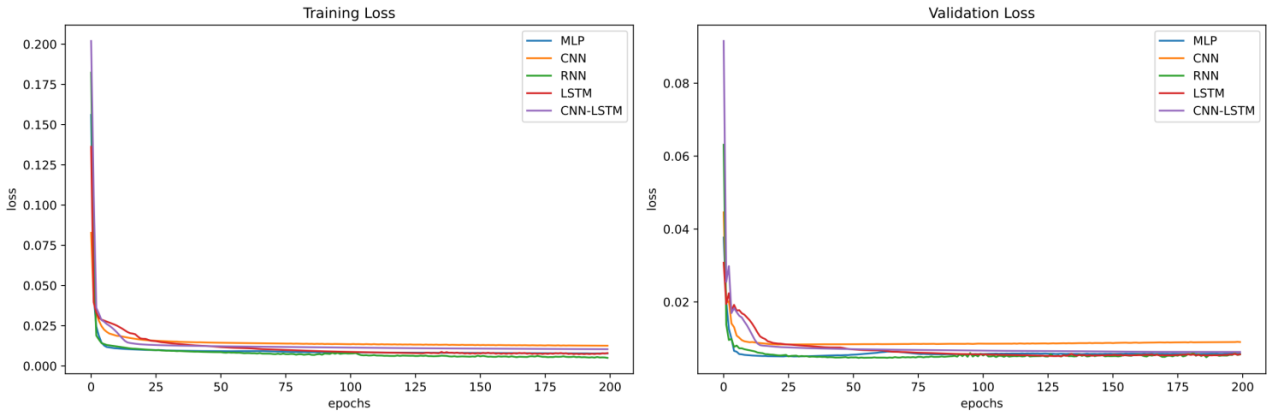


Figure S3 Training and validation loss curves (MSE) for five deep learning models over 200 epochs.


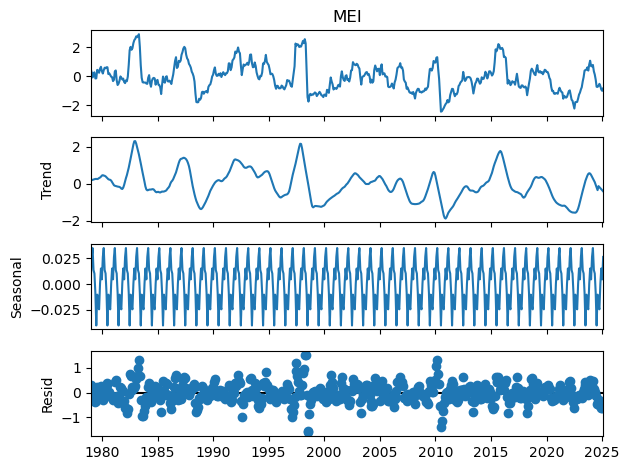


Figure S4 Seasonal decomposition of the MEI time series into trend, seasonal, and residual components.

Table S1. Summary of model types, spline basis functions, and knot configurations for region–host-specific GAMs

| Region-Host | Distribution | SelectedModel | Spline basis for *MEI* | Spline basis for *time* | No. of Knots (*time*) |
| --- | --- | --- | --- | --- | --- |
| East Asia-Domestic poultry | Negative binomial | Model 6 | TPRS | CRS | 40 |
| East Asia-Wild Birds | Negative binomial | Model 6 | CRS | TPRS | 10 |
| Southeast Asia-Domestic poultry | Negative binomial | Model 4 | NA | CRS | 15 |
| South Asia-Domestic poultry | Negative binomial | Model 6 | TPRS | TPRS | 10 |
| South Asia-Wild Birds | Poisson | Model 6 | TPRS | TPRS | 10 |
| West Asia-Domestic poultry | Negative binomial | Model 6 | TPRS | TPRS | 25 |
| West Asia-Wild Birds | Negative binomial | Model 6 | CRS | CRS | 5 |

Note: “TPRS” denotes thin plate regression spline; “CRS” denotes cubic regression spline; “NA” indicates that the variable *MEI* was modeled linearly. Knot selection followed a grid search strategy, as described in Section 2.2.2.2 of the Methods.

Table S2 Predicted monthly MEI values from March to August 2025 based on the RNN model.

| Year-Month | Predicted MEI |
| --- | --- |
| 2025-03 | -1.0 |
| 2025-04 | -1.1 |
| 2025-05 | -1.1 |
| 2025-06 | -1.0 |
| 2025-07 | -0.9 |
| 2025-08 | -0.8 |
